# Lipid Lowering Treatment and Eligibility for PCSK9 Inhibition in Post-Myocardial Infarction Patients in Italy: Insights from Two Contemporary Nationwide Registries

**DOI:** 10.1155/2020/3856242

**Published:** 2020-01-03

**Authors:** Furio Colivicchi, Michele Massimo Gulizia, Marcello Arca, Pier Luigi Temporelli, Lucio Gonzini, Vanessa Venturelli, Nuccia Morici, Ciro Indolfi, Domenico Gabrielli, Leonardo De Luca

**Affiliations:** ^1^Division of Cardiology, S. Filippo Neri Hospital, Roma, Italy; ^2^Division of Cardiology, Garibaldi-Nesima Hospital, Catania, Italy; ^3^Department of Internal Medicine and Medical Specialties, Sapienza University of Roma, Italy; ^4^Division of Cardiology, Istituti Clinici Scientifici Maugeri, IRCCS, Veruno (Novara), Italy; ^5^ANMCO Research Center, Fondazione per il Tuo cuore, Firenze, Italy; ^6^Division of Cardiology, S. Pertini Hospital, Roma, Italy; ^7^Division of Cardiology, Niguarda Ca'Grande Hospital, Milano, Italy; ^8^Cardiology Unit, Università degli Studi Magna Graecia, Catanzaro, Italy; ^9^Division of Cardiology, Augusto Murri Hospital, Fermo, Italy; ^10^Division of Cardiology, S. Giovanni Evangelista Hospital, Tivoli (Roma), Italy

## Abstract

**Introduction:**

The current use of lipid lowering therapies and the eligibility for proprotein convertase subtilisin/kexin-9 (PCSK9) inhibitors of patients surviving a myocardial infarction (MI) is poorly known.

**Methods:**

Using the data from two contemporary, nationwide, prospective, real-world registries of patients with stable coronary artery disease, we sought to describe the lipid lowering therapies prescribed by cardiologists in patients with a prior MI and the resulting eligibility for PCSK9 inhibitors according to the European Society of Cardiology (ESC)/European Atherosclerosis Society (EAS) and the Italian regulatory agency (Agenzia Italiana del Farmaco; AIFA) criteria. The study cohort was stratified according to the following low-density lipoprotein cholesterol (LDL-C) levels at the time of enrolment: <70 mg/dl; 70–99 mg/dl and ≥100 mg/dl.

**Results:**

Among the 3074 post-MI patients with LDL-C levels available, a target level of LDL-C < 70 mg/dl was present in 1186 (38.6%), while 1150 (37.4%) had LDL-C levels ranging from 70 to 99 mg/dl and the remaining 738 (24.0%) an LDL-C ≥ 100 mg/dl. A statin was prescribed more frequently in post-MI patients with LDL-C levels <70 mg/dl (97.1%) compared to the other LDL-C groups (*p* < 0.0001). A low dose of statin was prescribed in 9.3%, while a high dose in 61.4% of patients. Statin plus ezetimibe association therapy was used in less than 18% of cases. In the overall cohort, 293 (9.8%) and 450 (22.2%) resulted eligible for PCSK9 inhibitors, according to ESC/EAS and AIFA criteria, respectively.

**Conclusions:**

Post-MI patients are undertreated with conventional lipid lowering therapies. A minority of post-MI patients would be eligible to PCSK9 inhibitors according to ESC/EAS guidelines and Italian regulatory agency criteria.

## 1. Introduction

Although long-term prognosis of patients after a myocardial infarction (MI) has considerably improved, the residual risk of these patients remains high with a recurrence rate of ischemic fatal and nonfatal events of 20–30% within 3 years [1]. Several secondary prevention trials [2, 3] have consistently demonstrated a direct correlation between low-density lipoprotein cholesterol (LDL-C) levels achieved during lipid-lowering therapies and the risk of atherosclerotic cardiovascular disease (ASCVD). As a result, current international guidelines on the management of MI recommend decreasing LDL-C to a target level of <70 mg/dl using high-intensity statin therapy in combination with ezetimibe, if needed [4–6]. However, real-life data suggest that most post-MI patients fail to achieve the recommended targets [7, 8]. The reasons for poorly controlled LDL-C levels are underuse of lipid lowering therapies, lack of compliance to treatment or statin resistance and intolerance [9, 10].

The proprotein convertase subtilisin/kexin-9 (PCSK9) inhibitors evolocumab and alirocumab have emerged as a promising therapy for the treatment of hypercholesterolemia, since these agents are able to lower LDL-C by 50– 65% [11, 12]. Furthermore, two large outcomes trials [13, 14] have consistently demonstrated that both evolocumab and alirocumab are effective in reducing by 15% (*p* < 0.001) the recurrence of major adverse cardiovascular events in high risk patients with manifest ASCVD. Accordingly, guidelines for the use of PCSK9 inhibitors in patients at very high cardiovascular risk have been released by several scientific organizations. In particular, a joint consensus statement from the European Society of Cardiology (ESC) and European Atherosclerosis Society (EAS) suggested that PCSK9 use should be considered in patients with clinical ASCVD treated with maximal tolerated statin therapy and/or ezetimibe but still showing LDL-C >140 mg/dL (>3.6 mmol/L) or LDL-C >100 mg/dL (>2.6 mmol/L) in the absence/presence of indices of risk severity, such as familial hypercholesterolemia, diabetes mellitus or severe/extensive ASCVD [15]. On the other hand, in dealing with the potential financial impact of expensive PCSK9 inhibitors on health care systems, also national regulatory agencies have defined criteria for using these medications in clinical practice. In particular, the National Institute for Health and Care Excellence (NICE) recommended the prescription of PCSK9 inhibitors in ASCVD patients only if LDL-C concentration is persistently above 160 mg/dl (4.0 mmol/L) [16] and the Italian regulatory agency (Agenzia Italiana del Farmaco; AIFA) when LDL-C concentration remains above 100 mg/dL despite the use of maximally tolerated statin dose in combination with ezetimibe (http://www.agenziafarmaco.gov.it).

In light of the differences between the recommendations, no studies have compared the eligibility for PCSK9 inhibitors according to criteria of scientific societies or regulatory agencies. Analyses of large real-world database might be useful in order to provide this information, which is pivotal not only to estimate the subsequent budget impact associated with the widespread adoption of these therapies but also to evaluate the proportion of high risk ASCVD patients not reaching the recommended LDL-C targets who are deprived of benefit and improved outcomes by lack of use of PCSK9 inhibitors.

Using the data from the STable Coronary Artery Diseases RegisTry (START) [17] and the EYESHOT (EmploYEd antithrombotic therapies in patients with acute coronary Syndromes HOspitalized in iTaly) Post-MI [18], two Italian contemporary, nationwide registries on patients with stable coronary artery disease (CAD), we sought to describe the lipid lowering therapies prescribed in those with a prior MI and the resulting eligibility for PCSK9 inhibitors according to the criteria recommended by ESC/EAS and Italian regulatory agency.

## 2. Methods

The methods used to set up each registry have been described previously [17, 18]. Briefly, their primary objectives were to evaluate clinical characteristics as well as management and treatment of stable CAD patients admitted to Italian cardiology centres, using a catchment method broad enough to provide data representative of the entire country. Participation in the various registries had been offered to all Italian institutions. Physicians were instructed that participation in the registries should not affect their clinical care or patients' management. Informed consent was obtained from all patients who were aware of the nature and aims of the registries. Enrolment was made at the end of outpatient or day-hospital visit or at hospital discharge. Local Institutional Review Boards were informed of the study according to the Italian rules and approved the protocol.

In the START registry, 183 cardiology centers included 5070 consecutive patients with stable CAD in different periods of 3 months between March 2016 and February 2017 [17]. In the EYESHOT Post-MI registry, 165 cardiology centers included 1633 consecutive post-MI patients in different periods of 3 months between March and December 2017 [18].

In the present analysis we included only patients with a history of MI [ST-elevation MI (STEMI) and/or NonST elevation MI (NSTEMI)] and LDL cholesterol levels available at the time of enrolment.

To estimate the pre-treatment LDL cholesterol level, we multiplied the on-treatment LDL cholesterol level by a correction factor based on the potency of their LDL-C lowering regimen as suggested before [19]. In brief, we determined the estimated LDL cholesterol lowering potency of a specific lipid-lowering drug and dose. We multiplied the on-treatment LDL cholesterol level with that treatment potency, yielding an estimated pre-treatment LDL cholesterol level. In case of concomitant use of ezetimibe, we increased the relative LDL cholesterol reduction by 15% [20].

All patients included into the analysis were evaluated for PCSK9 inhibitor eligibility using the ESC/EAS clinical guidance recommendations [15] and the criteria released by the Italian regulatory agency AIFA. According to the ESC/EAS recommendations, any subject with documented clinical SCVD including post-MI patients without diabetes mellitus and with an LDL-C>140 mg/dl receiving maximally tolerated efficacious statin (preferably atorvastatin or rosuvastatin) in combination with ezetimibe or ezetimibe alone (statin intolerant) or post-MI diabetic patients with target organ damage and with an LDL-C >100 mg/dl while on maximally tolerated statin in combination with ezetimibe or ezetimibe alone (statin intolerant) were considered eligible for PCSK9 inhibitor therapy [15]. We considered diabetes mellitus as the only index of risk severity as the others (e.g. as familial hypercholesterolemia or the extension/rapid progression of ASCVD) were not available in both cohorts. According to the AIFA criteria, post-MI patients aged ≤80 years, estimated creatinine clearance (CrCl) ≥30 ml/min (according to the Cockroff-Gault equation) and LDL-C > 100 mg/dl despite treatment with high potency statins (20–40 mg rosuvastatin, 40–80 mg atorvastatin) plus ezetimibe or ezetimibe alone in the presence of a well-documented condition of statin intolerance. Were considered eligible for PCSK9 inhibitor therapy (http://www.agenziafarmaco.gov.it). As it was not possible to estimate the presence of statin intolerance, we have considered the use of ezetimibe alone as a proxy for statin intolerance. The study cohort was also stratified according to the following LDL-C levels at enrolment: <70 mg/dl; 70–99 mg/dl and ≥100 mg/dl.

### 2.1. Statistical Analysis

Categorical variables are presented as number and percentages and compared by the chi-square test. Continuous variables are presented as mean and standard deviation (SD), except for the statin dosages, which are reported as median and inter-quartile range (IQR) and were compared by the analysis of variance (ANOVA), if normally distributed, or by the Kruskall–Wallis test, if not. A *p* value <0.05 was considered statistically significant. All tests were 2-sided. Analyses were performed with SAS system software, version 9.4.

## 3. Results

Among the 6702 patients enrolled in the two registries, 3074 (45.9%) had a history of MI (STEMI and/or NSTEMI) and LDL-C levels available (2171 from the START and 903 from the EYESHOT Post-MI registry) and were considered in our analysis.

At the time of enrollment, a target level of LDL-C < 70 mg/dl was present in 1186 (38.6%) of post-MI patents enrolled, while 1150 (37.4%) had LDL-C levels ranging from 70 to 99 mg/dl and the remaining 738 (24.0%) an LDL-C ≥ 100 mg/dl. The mean LDL-C levels were 82.5 ± 32.3 mg/dl for the overall population and 54.1 ± 11.4, 82.9 ± 8.5 and 127.6 ± 26.4 mg/dl (*p* < 0.0001) for the 3 LDL-C groups, respectively. After adjustment for different statins and dosages, mean estimated pretreatment LDL-C values resulted as 105.9 ± 28.4, 162.8 ± 36.8 and 254.0 ± 82.5 mg/dl (*p* < 0.0001) in the three classes of patents, respectively.

Baseline characteristics of patients with different LDL-C levels at enrollment are shown in Table 1. Compared with the other groups, patients with LDL-C levels <70 mg/dl presented a significantly higher rate of prior revascularization, diabetes mellitus and hypertension compared to patients with higher cholesterol levels at enrollment. Patients with LDL-C levels on recommended target also presented lower values of total cholesterol, HDL-C and triglycerides and higher levels of creatinine and fasting glucose at enrollments as well as lower estimated pre-treatment LDL-C levels compared to other patient groups (Table 1).

### 3.1. Lipid-Lowering Agents Prescribed and Eligibility for PCSK9 Inhibitors

At the time of discharge or at the end of the visit, a statin was prescribed in 2928 (95.3%) post-MI patients. This occurred frequently in those with LDL-C levels <70 mg/dl (97.1%) compared to other groups (96.2% and 90.8% in those with 70–99 mg/dl and ≥100 mg/dl, respectively; *p* < 0.0001); on the other hand, less patients presenting LDL-C levels ≥100 mg/dl were receiving statin treatment (Table 2).

**Figure 1:**
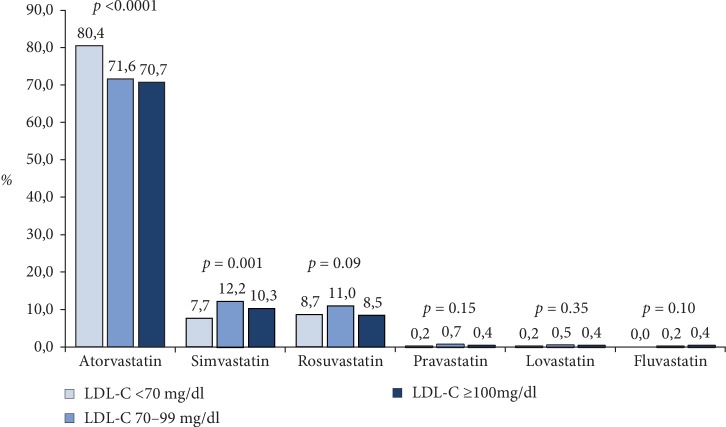
Statin compounds prescribed at the time of discharge/end of the visit in post-MI patients according to LDL-C levels.

**Figure 2:**
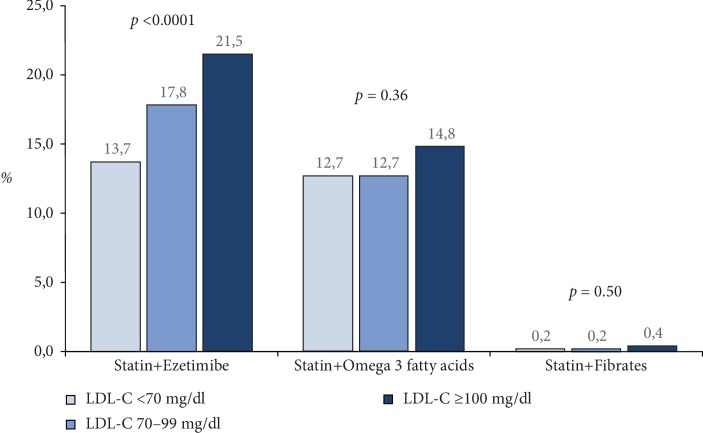
Associations of lipid lowering strategies^†^ in post-MI patients according to LDL-C levels. ^†^Other possible combinations not shown were used in less than 0.5% of cases.

A low dose of statin (atorvastatin ≤10 mg/day, fluvastatin ≤ 40 mg/day, lovastatin ≤ 20 mg/day, pravastatin ≤ 20 mg/day, rosuvastatin ≤ 5 mg/day or simvastatin ≤ 20 mg/day) was prescribed in 9.3%, while a high dose (atorvastatin ≥ 40 mg or rosuvastatin≥20 mg) was used in 61.4% of patients (Table 2). A significative difference was observed among the LDL-C groups for the statins dose (*p* = 0.0003). Atorvastatin was the most employed statin compound, especially among patients with LDL-C levels ≤ 70 mg/dl (Figure 1). However, the mean dosages of atorvastatin and simvastatin were higher in patients with LDL-C levels ≥100 mg/dl at enrolment compared to other groups (Table 2). Concerning the other lipid-lowering agents, ezetimibe alone (2.7% vs. 0.8% and 1.0%, respectively; *p* = 0.0009), and the association of statin+ezetimibe was more frequently used in patients with LDL-C levels ≥100 mg/dl compared to the other groups (*p* < 0.0001) (Figure 2), even when high dose statins and ezetimibe was considered (11.9% vs. 6.2% and 8.3%, respectively; *p* < 0.0001). On the other hand, fibrates and omega-3 fatty acids in association with statins was prescribed in about 13% of patients and this prevalence was comparable among the 3 groups (Figure 2).

In the overall post-MI cohort treated with statins and/or ezetimibe (*n* = 2977), 293 (9.8%) resulted eligible for PCSK9 inhibitors according to ESC/EAS criteria (Table 3). Considering the 2029 patients treated with high-dose statins plus ezetimibe and creatinine levels available, 450 (22.2%) were eligible to PCSK9 inhibition following AIFA criteria (Table 3).

## 4. Discussion

The major results of present analysis including a large, contemporary, real world cohort of post-MI were the following: (1) about 40% of patients reached the target of LDL-C levels recommended by international guidelines; (2) although statins are prescribed in the majority of patients with a history of MI, a high dose is employed in 61.4% of cases and the association therapy is underused; (3) only a minority of these patients at very high risk are eligible for PCSK9 inhibitors according to current European or national criteria.

For patients at very high risk, an LDL-C goal of <70 mg/dl or a reduction of at least 50% if the baseline LDL-C is between 70 and 135 mg/dl is a Class I recommendation [4–6]. Data from registries show that only 20–40% of patients with a recent MI or with stable coronary artery disease receiving statins attain recommended LDL-C goals [21–23]. Our findings are in agreement with these reports also indicating that patients on LDL-C target presented a higher rate of risk factors such as prior revascularization, diabetes mellitus, and hypertension compared to patents not on target. This might suggest that cardiologists tend to treat more aggressively patients who are perceived as to be at higher risk (for the presence of additional risk factors or for a more severe clinical presentation of ASCVD). However, the lack of association between the use of high potency statins and the achieved LDL-C levels, might, alternatively, indicate that patients on target show less severe baseline LDL-C elevation. The lower estimated pretreatment LDL-C values in the group of patients on target are in line with this possibility. Nevertheless, this latter observation further indicates that in the real word clinical setting there is a little effort in adjusting type and intensity of LDL-lowering treatment to the distance to be filled between basal and target LDL-C values.

Several studies have suggested improvements in use and adherence to statin therapy following an MI over the past 2 decades [24–26]. In a large retrospective cohort study of adults who initiated statins in 2007–2014, the adherence to statin therapy reached 64% at 1 year after a MI [27]. In another recent analysis of more than 110.000 hospitalizations for MI in United States, the use of high-intensity statin therapy increased progressively between 2011 and 2014 [28]. Indeed, by the end of 2014, the majority of patients discharged following hospitalization for MI filled high intensity doses. Notably, the evaluation of type of statin usage indicated that the most commonly prescribed statin shifted from simvastatin to atorvastatin from the first quarter of 2011–2014 [28]. Accordingly, in our series a high intensity statin was prescribed in 61.4% of the overall population and atorvastatin was the most prescribed statin compound, especially among patients with LDL-C levels below the recommended target levels. However, we have observed that lipid lowering association therapies are less used being the combination statin plus ezetimibe prescribed in about 1 over 5 patients.

Several guidelines from scientific societies have indicate that PCSK9 inhibitors should be considered in high risk ASCVD patients if LDL-C goals are not reached with conventional LDL-lowering therapies [4–6]. Indeed, according to the ESC/EAS joint document, patients should be titrated to the maximally tolerated dose of efficacious statin (preferably atorvastatin or rosuvastatin); if LDL-C levels are still above recommended goals, addition of ezetimibe is recommended before consideration of a PCSK9 inhibitor in order to ensure appropriate patient pre-treatment before prescription of new drugs [15]. Even the 2016 American College of Cardiology (ACC) expert consensus decision pathway on the role of nonstatin therapies for LDL-C lowering in the management of atherosclerotic cardiovascular disease recommend the use of ezetimibe prior to considering PCSK9 inhibitors [29]. In general, the ESC/EAS criteria appear to be more conservative than the North American criteria [29]. A recent analysis of a prospective Swiss cohort of 2023 patients hospitalized for acute coronary syndromes between 2009 and 2014 indicated that recommendations issued by the ACC guidelines would lead to 5-fold higher eligibility rates for PCSK9 inhibitors as compared to the ESC/EAS consensus statement at 1 year (13.4% vs. 2.7%, respectively) [30], simulating a fixed effect of ezetimibe. Not considering the simulated effect of ezetimibe, the rate of patients eligible for PCSK9 inhibitors increased to 11% according to ESC/EAS criteria [30]. This rate is close to what we observed in our cohort, in whom ezetimibe was used in a minority of cases and the rate of eligibility to PCSK9 inhibitors resulted as 10% by applying the ESC/EAS recommendations. On the other hand, very few systematic evaluations of rates of eligibility for PCSK9 inhibitors by using regulatory criteria have been reported so far. In a recent survey where records of 596 patients with cardiovascular diseases in two large hospitals in Liverpool were analyzed, it was estimated that 2.2% of post-MI patients were eligible under the current guidance of NICE lipid targets criteria [16]. In our cohort, we found that 22% of post-MI met the AIFA criteria and this figure was about double compared to that obtained applying the ESC/EAS criteria and ten times higher than that reported under the NICE criteria [16]. Therefore, our analysis definitively confirmed that, as a whole, the ESC/EAS recommendations are more conservative than AIFA recommendations. Although decisions to use these medications must be tempered by the financial constraints of particular healthcare systems, ESC/EAS recommendation may exclude a significant proportion of very high-risk patients from clinical benefit of PCSK9 inhibitors treatment. Of note, in these patients – namely those with a prior hard cardiovascular event-the 10-year rate of major cardiovascular events is about 45%, even if treated with maximally tolerated statins [5]. The FOURIER study demonstrated that treatment with evolocumab for those with an LDL-C > 100 (the AIFA threshold) should achieve an absolute risk reduction of about 0.6%/year [13].

### 4.1. Study Limitations

Our study must be evaluated in the light of some limitations. First, we were not able to assess the LDL-C in 39% of patients included in the 2 studies, therefore the actual rate of eligibility for PCSK9 inhibitors could be underestimated. In addition, in modeling the impact of EAS criteria we considered all statins at any dosage as well as monotherapy with ezetimibe and this might have included patients who are responsive to high-potency statins in combination with ezetimibe. It must be acknowledged that a more restrictive definition of statin background therapy would determine a further reduction of proportion of patients eligible under the ESC/EAS criteria. More, creatinine levels, needed for the assessment of eligibility according to the AIFA criteria, were not available in 235 (7.6%) of our study cohort. However, to the best of our knowledge, this is the largest prospective cohort of post-MI patients in whom the eligibility for PCSK9 inhibitors has been assessed. Second, data reported in the present analysis are limited to the time of enrolment and we do not have data on long-term persistence to prescribed therapies, their changes and relative outcomes. Nevertheless, a clinical follow-up at 1 year from enrolment of one of the considered registries (START study) has been published, showing a persistence to statin therapy higher than 90%. Finally, even if the participating centers were asked to include in the registry all consecutive patients admitted with prior MI, we were not able to verify the enrolment process, due to the absence of administrative auditing.

## 5. Conclusions

The findings observed in this large cohort of post-MI patients managed by cardiologists are consistent with current literature on their suboptimal treatment in real-world settings and provides further evidence that large proportions of these patients would benefit from more aggressive treatment with conventional lipid lowering therapies. Less than 10% and 30% of post-MI patients would be eligible to PCSK9 inhibitors according to ESC/EAS guidelines and Italian regulatory agency criteria, respectively.

## Figures and Tables

**Table 1:** Clinical characteristics, hemodynamic and laboratory finding of patients enrolled according to baseline LDL-C levels.

	Total (*n*. 3074)	LDL-C < 70 mg/dl (*n*. 1186)	LDL-C 70–99 mg/dl (*n*. 1150)	LDL-C ≥ 100 mg/dl (*n*. 738)	*p*
Age, mean ± SD	66 ± 11	66 ± 11	66 ± 11	66 ± 11	0.31
Female, *n* (%)	584 (19.0)	195 (16.4)	222 (19.3)	167 (22.6)	0.003
Previous STEMI, *n* (%)	1625 (52.9)	641 (54.1)	611 (53.1)	373 (50.5)	0.32
Previous NSTE-ACS, *n* (%)	1575 (51.2)	590 (49.8)	592 (51.5)	393 (53.3)	0.32
Prior coronary revasculariz, *n* (%)	2696 (87.7)	1089 (91.8)	1000 (87.0)	607 (82.3)	<0.0001
BMI, mean ± SD	27.4 ± 4.1	27.4 ± 4.1	27.4 ± 4.0	27.3 ± 4.2	0.89
Current smokers, *n* (%)	609 (19.8)	205 (17.3)	216 (18.8)	188 (25.5)	<0.0001
Diabetes, *n* (%)	841 (27.4)	371 (31.3)	293 (25.5)	177 (24.0)	0.0004
Hypertension, *n* (%)	2369 (77.1)	943 (79.5)	869 (75.6)	557 (75.5)	0.04
History of heart failure, *n* (%)	422 (13.7)	176 (14.8)	156 (13.6)	90 (12.2)	0.26
History of atrial fibrillation, *n* (%)	368 (12.0)	143 (12.1)	130 (11.3)	95 (12.9)	0.59
Peripheral artery disease, *n* (%)	222 (7.2)	79 (6.7)	93 (8.1)	50 (6.8)	0.36
History of stroke/TIA, *n* (%)	134 (4.4)	49 (4.1)	53 (4.6)	32 (4.3)	0.85
Chronic kidney disease, *n* (%)	360 (11.7)	162 (13.7)	118 (10.3)	80 (10.8)	0.03
Severe liver disease, *n* (%)^∗^	21 (0.7)	10 (0.8)	3 (0.3)	8 (1.1)	0.07
COPD, *n* (%)	352 (11.5)	134 (11.3)	126 (11.0)	92 (12.5)	0.59
Malignancy, *n* (%)	144 (4.7)	52 (4.4)	60 (5.2)	32 (4.3)	0.56
SBP, mean ± SD	129 ± 17	128 ± 17	130 ± 16	130 ± 18	0.02
DBP, mean ± SD	77 ± 9	76 ± 9	77 ± 9	77 ± 10	0.12
HR, mean ± SD	66 ± 11	66 ± 11	65 ± 11	67 ± 12	0.006
Ejection fraction (%), mean ± SD	53 ± 10	53 ± 10	54 ± 10	53 ± 10	0.09
available for 2901 pts					
Cholesterol (mg/dL), mean ± SD	149.9 ± 37.5	120.6 ± 20.7	149.8 ± 19.0	197.1 ± 31.2	<0.0001
available for 2917 pts					
HDL-C (mg/dL), mean ± SD	45.8 ± 14.1	44.8 ± 15.3	45.9 ± 12.9	47.2 ± 13.9	<0.0001
available for 2810 pts					
Triglycerides (mg/dL), mean ± SD	121.9 ± 60.9	113.5 ± 57.2	118.6 ± 54.7	140.8 ± 71.0	<0.0001
available for 2853 pts					
Haemoglobin (g/dL), mean ± SD	13.7 ± 1.7	13.6 ± 1.8	13.8 ± 1.7	13.8 ± 1.6	0.0004
available for 2820 pts					
Creatinine (mg/dL), mean ± SD	1.1 ± 0.5	1.1 ± 0.6	1.0 ± 0.5	1.0 ± 0.4	0.03
available for 2839 pts					
eCrCl, mean ± SD	80.1 ± 26.5	79.3 ± 25.8	79.9 ± 23.8	81.7 ± 31.0	0.43
available for 2839 pts					
Fasting glucose (mg/dL), mean ± SD	112.1 ± 34.8	114.4 ± 36.9	111.6 ± 33.4	109.1 ± 33.2	0.004
available for 2649 pts					

STEMI, ST elevation myocardial infarction; NSTEMI, non ST elevation myocardial infarction; CS, acute coronary syndrome; TIA, transient ischemic attack; COPD, chronic obstructive pulmonary disease; SBP, systolic blood pressure; DBP, diastolic blood pressure; HR, heart rate; HDL-C, high density lipoprotein cholesterol; eCrCl, estimated creatinine clearance;

^∗^in presence of ascites, variceal haemorrhage and/or hepatic encephalopathy.

**Table 2:** Pharmacological therapy at discharge/end visit according to baseline LDL-C levels.

	Total (*n*. 3074)	LDL-C < 70 mg/dl (*n*. 1186)	LDL-C 70–99 mg/dl (*n*. 1150)	LDL-C ≥ 100 mg/dl (*n*. 738)	*p*
Oral anticoagulants, *n* (%)	288 (9.4)	110 (9.3)	98 (8.5)	80 (10.8)	0.24
ASA, *n* (%)	2785 (90.6)	1086 (91.6)	1050 (91.3)	649 (87.9)	0.02
Thienopyridine, ^§^*n* (%)	1597 (52.0)	614 (51.8)	556 (48.4)	427 (57.9)	0.0003
ASA/thienopyridine, *n* (%)	2946 (95.8)	1140 (96.1)	1103 (95.9)	703 (95.3)	0.64
DAPT (ASA + thienopyridine), *n* (%)	1436 (46.7)	560 (47.2)	503 (43.7)	373 (50.5)	0.01
ACE-inhibitors, *n* (%)	1735 (56.4)	694 (58.5)	635 (55.2)	406 (55.0)	0.18
ARBs, *n* (%)	690 (22.5)	269 (22.7)	274 (23.8)	147 (19.9)	0.14
ACE/ARBs	2405 (78.2)	958 (80.8)	902 (78.4)	545 (73.9)	0.002
Beta-blockers, *n* (%)	2457 (79.9)	976 (82.3)	914 (79.5)	567 (76.8)	0.01
Mineralcorticoid antagonists (MRAs), *n* (%)	334 (10.9)	138 (11.6)	123 (10.7)	73 (9.9)	0.48
Statins, *n* (%)	2928 (95.3)	1152 (97.1)	1106 (96.2)	670 (90.8)	<0.0001
Low-dose statins	286 (9.3)	105 (8.9)	130 (11.3)	51 (6.9)	0.005
High-intensity statins	1888 (61.4)	753 (63.5)	690 (60.0)	445 (60.3)	0.17
*Dose of statins (mg), mean SD)*					
Atorvastatin	40 [40-40]	40 [20–40]	40 [40-40]	40 [40–80]	0.0009
Fluvastatin	80 [80-80]	—	45 [10–80]	80 [80-80]	0.41
Lovastatin	20 [20–40]	30 [20–40]	20 [20–40]	40 [20–40]	0.66
Pravastatin	40 [40-40]	40 [40-40]	40 [40-40]	40 [40-40]	0.73
Rosuvastatin	15 [10–20]	10 [10–20]	20 [10–20]	20 [10–20]	0.09
Simvastatin	20 [20–40]	20 [20–40]	20 [20–40]	40 [20–40]	0.02

ASA, aspirin; DAPT, dual antiplatelet therapy, ACE angiotensin converting enzyme; ARB, angiotensin receptor blockers.

^§^Clopidogrei/prasugrel/ticagrelor/ticlopidina.

**Table 3:** Estimated prevalence of post-MI patients eligible to PCSK9 inhibitors according to the EAS/ESC and AIFA criteria.

EAS/ESC criteria (*n* = 2977)	*N*	%
Statin and/or ezetimibe^†^ + noDM + LDL-C >140 mg/dl	130	4,37
Statins and/or ezetimibe^†^ + DM + LDL-C >100 mg/dl	163	5.48

AIFA criteria (*n* = 2029)		

High intensity statins and ezetimbe^‡^ + age ≤80 yrs + eCrCl ≥ 30 ml/min + LDL-C >100 mg/dl	450	22.2

DM, diabetes mellitus; eCrCl, estimated creatinine clearance, LDL-C, low density lipoprotein cholesterol.

^†^These groups included patients receiving any statins at any dosage. Monoterapy with ezetimibe has been taken as a proxy of statin intolerance.

^‡^High intensity statins have been considered atorvastatin 40–80 mg /day or rosuvastatin 20–40 mg/day. Monoterapy with ezetimibe has been taken as a proxy of statin intolerance.

## Data Availability

The data used to support the findings of this study are available at the ANMCO Centro Studi that is the data owner.

## References

[1] Jernberg T., Hasvold P., Henriksson M., Hjelm H., Thuresson M., Janzon M. (2015). Cardiovascular risk in post-myocardial infarction patients: nationwide real world data demonstrate the importance of a long-term perspective. *European Heart Journal*.

[2] Baigent C., Blackwell L., Emberson J. (2010). Efficacy and safety of more intensive lowering of LDL cholesterol: a meta-analysis of data from 170,000 participants in 26 randomised trials. *The Lancet*.

[3] Cannon C. P., Blazing M. A., Giugliano R. P. (2015). Ezetimibe added to statin therapy after acute coronary syndromes. *New England Journal of Medicine*.

[4] Roffi M., Patrono C., Collet J. P. (2016). 2015 ESC guidelines for the management of acute coronary syndromes in patients presenting without persistent ST-segment elevation: task force for the management of acute coronary syndromes in patients presenting without persistent ST-segment elevation of the European society of cardiology (ESC). * European Heart Journal*.

[5] Catapano A. L., Graham I., De Backer G. (2016). 2016 ESC/EAS guidelines for the management of dyslipidaemias: the task force for the management of dyslipidaemias of the European society of cardiology (ESC) and European Atherosclerosis Society (EAS) developed with the special contribution of the European assocciation for cardiovascular prevention & Rehabilitation (EACPR). *Atherosclerosis*.

[6] Stone N. J., Robinson J. G., Lichtenstein A. H. (2014). 2013 ACC/AHA guideline on the treatment of blood cholesterol to reduce atherosclerotic cardiovascular risk in adults: a report of the American College of Cardiology/American Heart Association task force on practice guidelines. *Circulation*.

[7] Hirsh B. J., Smilowitz N. R., Rosenson R. S., Fuster V., Sperling L. S. (2015). Utilization of and adherence to guideline-recommended lipid-lowering therapy after acute coronary syndrome: opportunities for improvement. *Journal of the American College of Cardiology*.

[8] Penning-van Beest F. J., Termorshuizen F., Goettsch W. G., Klungel O. H., Kastelein J. J., Herings R. (2007). Adherence to evidence-based statin guidelines reduces the risk of hospitalizations for acute myocardial infarction by 40%: a cohort study. *European Heart Journal*.

[9] Schultz J. S., O’Donnell J. C., McDonough K. L., Sasane R., Meyer J. (2005). Determinants of compliance with statin therapy and low-density lipoprotein cholesterol goal attainment in a managed care population. *The American Journal of Managed Care*.

[10] Chan D. C., Shrank W. H., Cutler D. (2010). Patient, physician, and payment predictors of statin adherence. *Medical Care*.

[11] Navarese E. P., Kolodziejczak M., Schulze V. (2015). Effects of proprotein convertase subtilisin/kexin type 9 antibodies in adults with hypercholesterolemia: a systematic review and meta-analysis. *Annals of Internal Medicine*.

[12] Lipinski M. J., Benedetto U., Escarcega R. O. (2016). The impact of proprotein convertase subtilisin-kexin type 9 serine protease inhibitors on lipid levels and outcomes in patients with primary hypercholesterolaemia: a network meta-analysis. *European Heart Journal*.

[13] Sabatine M. S., Giugliano R. P., Keech A. C. (2017). Evolocumab and clinical outcomes in patients with cardiovascular disease. *New England Journal of Medicine*.

[14] Schwartz G. G., Steg P. G., Szarek M. (2018). Alirocumab and cardiovascular outcomes after acute coronary syndrome. *New England Journal of Medicine*.

[15] Landmesser U., John Chapman M., Farnier M. (2017). European Society of Cardiology/European Atherosclerosis Society task force consensus statement on proprotein convertase subtilisin/kexin type 9 inhibitors: practical guidance for use in patients at very high cardiovascular risk. *European Heart Journal*.

[16] Elamin A. F. M., Grafton-Clarke C., Wen Chen K. (2019). Potential use of PCSK9 inhibitors as a secondary preventative measure for cardiovascular disease following acute coronary syndrome: a UK real-world study. *Postgraduate Medical Journal*.

[17] De Luca L., Temporelli P. L., Lucci D. (2018). Current management and treatment of patients with stable coronary artery diseases presenting to cardiologists in different clinical contexts: a prospective, observational, nationwide study. *European Journal of Preventive Cardiology*.

[18] De Luca L., Piscione F., Colivicchi F. (2018). Contemporary management of patients referring to cardiologists one to three years from a myocardial infarction: the EYESHOT post-MI study. *International Journal of Cardiology*.

[19] Law M. R., Wald N. J., Rudnicka R. (2003). Quantifying effect of statins on low density lipoprotein cholesterol, ischaemic heart disease, and stroke: systematic review and meta-analysis. *BMJ*.

[20] Davidson M. H., Ballantyne C. M., Kerzner B. (2004). Efficacy and safety of ezetimibe coadministered with statins: randomised, placebo-controlled, blinded experience in 2382 patients with primary hypercholesterolemia. *International Journal of Clinical Practice*.

[21] Gencer B., Auer R., Nanchen D. (2015). Expected impact of applying new 2013 AHA/ACC cholesterol guidelines criteria on the recommended lipid target achievement after acute coronary syndromes. *Atherosclerosis*.

[22] Gitt A. K., Drexel H., Feely J. (2012). Persistent lipid abnormalities in statin-treated patients and predictors of LDL-cholesterol goal achievement in clinical practice in Europe and Canada. *European Journal of Preventive Cardiology*.

[23] Reiner Ž., De Backer G., Fras Z. (2016). EUROASPIRE Investigators. Lipid lowering drug therapy in patients with coronary heart disease from 24 European countries—findings from the EUROASPIRE IV survey. *Atherosclerosis*.

[24] Choudhry N. K., Setoguchi S., Levin R., Winkelmayer W. C., Shrank W. H. (2008). Trends in adherence to secondary prevention medications in elderly post-myocardial infarction patients. *Pharmacoepidemiology and Drug Safety*.

[25] Colantonio L. D., Huang L., Monda K. L. (2017). Adherence to high-intensity statins following a myocardial infarction hospitalization among medicare beneficiaries. *JAMA Cardiology*.

[26] Ferrières J., Rouyer M. V., Lautsch D. (2016). Improvement in achievement of lipid targets in France: comparison of data from coronary patients in the DYSIS and DYSIS II studies. *International Journal of Cardiology*.

[27] Colantonio L. D., Rosenson R. S., Deng L. (2019). Adherence to statin therapy among US adults between 2007 and 2014. *Journal of the American Heart Association*.

[28] Rosenson R. S., Farkouh M. E., Mefford M. (2017). Trends in use of high-intensity statin therapy after myocardial infarction, 2011 to 2014. *Journal of the American College of Cardiology*.

[29] Lloyd-Jones D. M., Morris P. B., Ballantyne C. M. (2016). 2016 ACC expert consensus decision pathway on the role of nonstatin therapies for LDL-cholesterol lowering in the management of atherosclerotic cardiovascular disease risk: a report of the American college of cardiology task force on clinical expert consensus documents. *Journal of the American College of Cardiology*.

[30] Gencer B., Koskinas K. C., Räber L. (2017). Eligibility for PCSK9 inhibitors according to American College of Cardiology (ACC) and European Society of Cardiology/European Atherosclerosis Society (ESC/EAS) guidelines after acute coronary syndromes. *Journal of the American Heart Association*.

